# The importance of increasing population diversity in genetic studies of type 2 diabetes and related glycaemic traits

**DOI:** 10.1007/s00125-021-05575-4

**Published:** 2021-09-30

**Authors:** Inês Barroso

**Affiliations:** Exeter Centre of Excellence for Diabetes research (EXCEED), University of Exeter Medical School, Exeter, UK

**Keywords:** Genetics, Genome-wide association study (GWAS), Meta-analysis, Multi-ancestry, Polygenic risk score (PRS), Precision medicine, Review

## Abstract

Type 2 diabetes has a global prevalence, with epidemiological data suggesting that some populations have a higher risk of developing this disease. However, to date, most genetic studies of type 2 diabetes and related glycaemic traits have been performed in individuals of European ancestry. The same is true for most other complex diseases, largely due to use of ‘convenience samples’. Rapid genotyping of large population cohorts and case–control studies from existing collections was performed when the genome-wide association study (GWAS) ‘revolution’ began, back in 2005. Although global representation has increased in the intervening 15 years, further expansion and inclusion of diverse populations in genetic and genomic studies is still needed. In this review, I discuss the progress made in incorporating multi-ancestry participants in genetic analyses of type 2 diabetes and related glycaemic traits, and associated opportunities and challenges. I also discuss how increased representation of global diversity in genetic and genomic studies is required to fulfil the promise of precision medicine for all.

**Graphical abstract:**

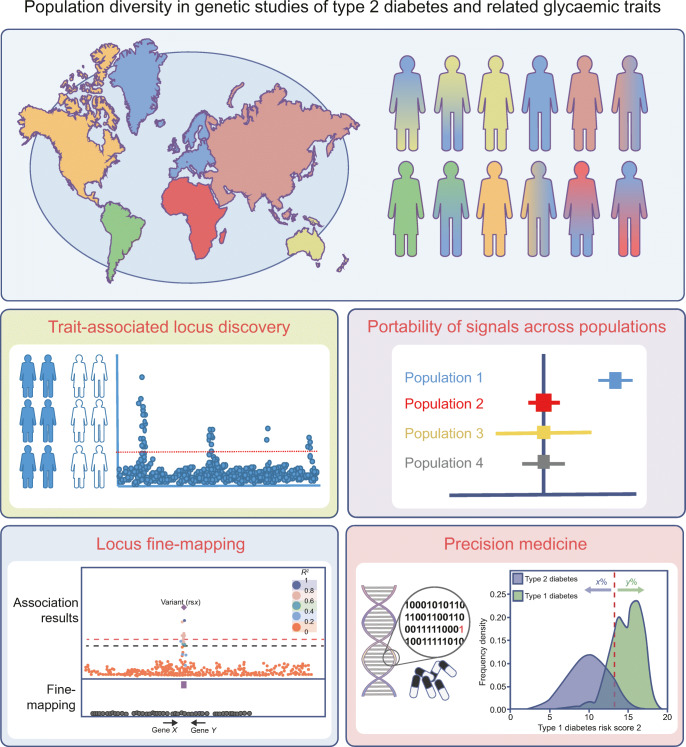

**Supplementary Information:**

The online version contains a slideset of the figures for download available at 10.1007/s00125-021-05575-4.

## Introduction

Type 2 diabetes is a multifactorial disease where a combination of genes, lifestyle and environment contribute to disease predisposition worldwide. The IDF projected that by 2045, 700 million people worldwide would have diabetes, with low- and middle-income countries accounting for the largest increases in prevalence [1].

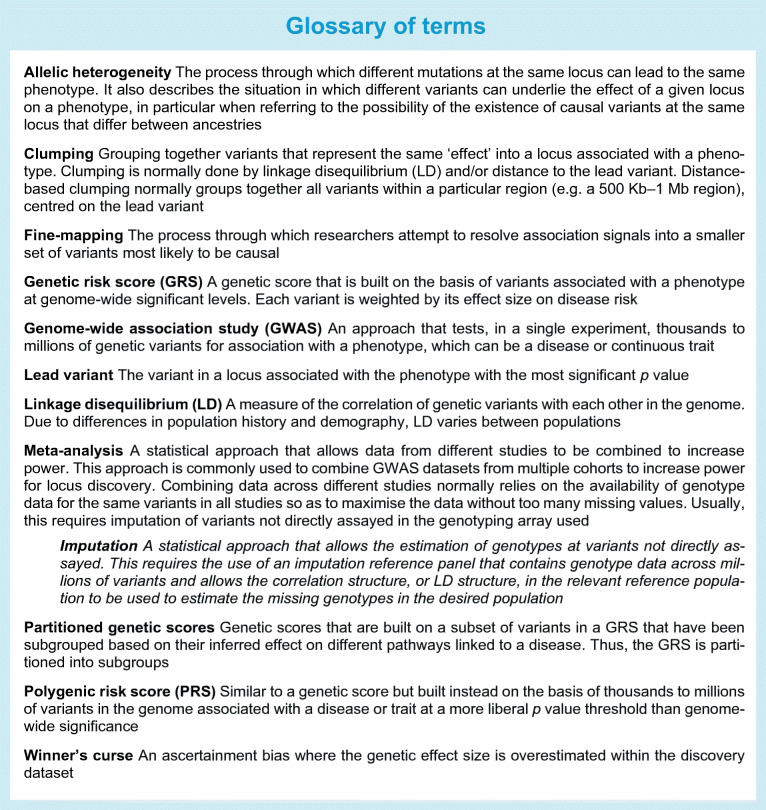



Since 2005, with the advent of genome-wide association studies (GWAS), the number of genetic loci known to influence type 2 diabetes risk and/or related quantitative glycaemic measures (e.g. glucose, insulin, HbA_1c_ levels) has surged. To date, more than 270 loci (with >400 signals) associated with type 2 diabetes risk and/or glycaemic traits have been identified mostly through meta-analysis of existing GWAS [2–6]. Despite this success, most type 2 diabetes GWAS do not represent the diversity of affected individuals as they have focused on individuals of European ancestry [7, 8] and, more recently, East Asian ancestry [2, 3]. This means we are still missing important aetiological factors that may differ among diverse global populations and consequently we may be increasing health disparities. In healthcare, sociocultural self-reported ethnicity or ‘race’ are often used as proxies for genetic ancestry. This is particularly problematic, as these proxies are confounded by socioeconomic status and cultural and lifestyle factors, and do not consider the genetic heterogeneity between individuals of the same self-reported ethnicity [9]. Different self-reported ethnic groups overlap genetically and two individuals of the same self-reported ethnicity may be genetically more ‘distant’ from one another than two individuals that each identify with a different ethnic group. This review aims to highlight the opportunities and challenges of including datasets from a broad range of population ancestries in genetic studies of type 2 diabetes and related traits. It also discusses how increasing diversity in genetic studies may impact on precision medicine in type 2 diabetes.

## GWAS from diverse populations

The lack of diversity in GWAS has been well documented [10–13] and has spurred new efforts to increase global representation, including the Human Health and Hereditary initiative in Africa [14], the All of Us programme in the USA [15] and new initiatives such as the ‘Latin American Alliance for Genomic Diversity’ (International Common Disease Alliance [ICDA], plenary programme 2020), the Taiwan precision medicine initiative [16] and The Brazilian Initiative on Precision Medicine (BIPMed) [17].

### Allelic frequency differences between populations aid locus discovery

GWAS in diverse populations has facilitated the discovery of novel type 2 diabetes aetiological factors owing to their divergent allele frequency across populations. One example is the risk haplotype near *SLC16A11*, discovered in Mexicans, that has high frequency in populations from the Americas (~50%), intermediate frequency in East Asians (~10%) and is rare or absent in populations from Europe and Africa [18]. Other examples include the rare Glu508Lys variant in *HNF1A* identified in Latinos that increases type 2 diabetes risk fivefold [19] and the East Asian Arg193His *PAX4* variant [20].

The largest analyses of type 2 diabetes in African Americans to date identified novel African American signals at *HLA-B* and *INS-IGF2* loci [21]. GWAS of cardiometabolic traits including African participants are still few [22–26] but further highlight a type 2 diabetes risk variant at *ZRANB3*, which is monomorphic elsewhere [24], and new African signals at *TCF7L2* (rs17746147) and near *AGMO* (rs73284431 [23]). A pan-African GWAS of 34 cardiometabolic traits that included 14,126 individuals identified a variant driven by the α^−3.7^ thalassaemia deletion associated with HbA_1c_ in Ugandans [25]. This deletion is more frequent in Ugandans as it confers resistance to severe malaria, which is endemic in Uganda [25].

These are important examples of population-specific signals (i.e. signals where the variant is very rare or monomorphic outside the cognate population, or signals where the effect of the variant on the trait has not been observed outside those cognate populations). Nonetheless, they can reveal population-specific disease aetiology, provide novel insights into pathophysiological pathways involved in disease and highlight novel aspects of biology not previously understood.

### Population-specific signals may be clinically important

Population-specific signals can identify variants that have large effects in cognate populations and, hence, may have an important translational impact in those populations. For example, the *TBC1D4* nonsense variant p.Arg684ter was initially found in Inuits from Greenland [27], where it has a high prevalence (17%) and large effect size (homozygous carriers have an approximately tenfold increased risk of type 2 diabetes), but is very rare or absent elsewhere. The same variant has now been detected at high frequency (~13–16% minor allele frequency) in North American Inuit populations. Here, it was shown that unless postprandial glucose levels were tested, 32% of *TBC1D4* p.Arg684ter carriers with prediabetes (defined as fasting plasma glucose 5.6–6.9 mmol/l, 2 h 75 g OGTT plasma glucose 7.8–11.0 mmol/l and/or HbA_1c_ 5.7–6.4% [39–46 mmol/mol]) and diabetes would remain undiagnosed [28]. In light of increasing diabetes prevalence in the Inuit [29], it has been suggested that stratifying diabetes diagnoses based on an individual’s *TBC1D4* p.Arg684ter genotype, and performing OGTTs in carriers of this variant, may be appropriate in this population [28]. In addition, *TBC1D4* acts on the insulin-stimulated glucose response pathway so it is plausible that carriers for this variant will have improved response to insulin sensitisers, although clinical trials have yet to be performed to test this [28]. On the other hand, a recent longitudinal analysis of Inuits in Greenland suggested that homozygosity for *TBC1D4* p.Arg684ter did not significantly increase risk of incident CVD in this population [30]. Given the small number of homozygous *TBC1D4* p.Arg684ter individuals in the study (*n* = 142), the possible inaccuracy in defining CVD outcomes, insufficient number of follow-up years, or other factors discussed by the authors [30], it is critical to replicate this finding. If replicated, this could suggest that diabetes associated with homozygosity for *TBC1D4* p.Arg684ter is similar to MODY due to *GCK* mutations [31], and would impact on how diabetes is managed in individuals homozygous for *TBC1D4* p.Arg684ter. Overall, this example highlights the potential importance of capturing population-specific signals for precision medicine approaches in diabetes diagnosis.

### Interpreting population-specific signals can be challenging

Establishing the broader relevance and reproducibility of population-specific signals, especially those that result from sequence-based rare variant analysis, can be difficult. This is because due to founder effects, drift and selection, population isolates are enriched for alleles that may be very rare or absent elsewhere [32]. In addition, some indigenous specific variants originate from discovery sample sizes in the order of thousands rather than hundreds of thousands and large population resources for replication are not always readily available. Naturally, larger effect sizes in these population-specific signals are not uncommon, as these are the effect sizes some of these smaller discovery samples are well-powered to detect. In these scenarios, given the high multiple testing burden, the lower power and the absence of replication datasets, it can be hard to distinguish between true population-specific signals and false-positive associations.

## Genome-wide multi-ancestry genetic analyses

Recently, efforts to jointly analyse different genetic datasets from populations of diverse ancestry have become more widespread [5, 6]. These multi-ancestry genetic analyses boost power for new locus discovery, provide the opportunity to test for widespread replication of signals across independent populations and allow exploration of the genetic architecture of phenotypes across ancestries.

### Portability of signals across populations

**Fig. 1:**
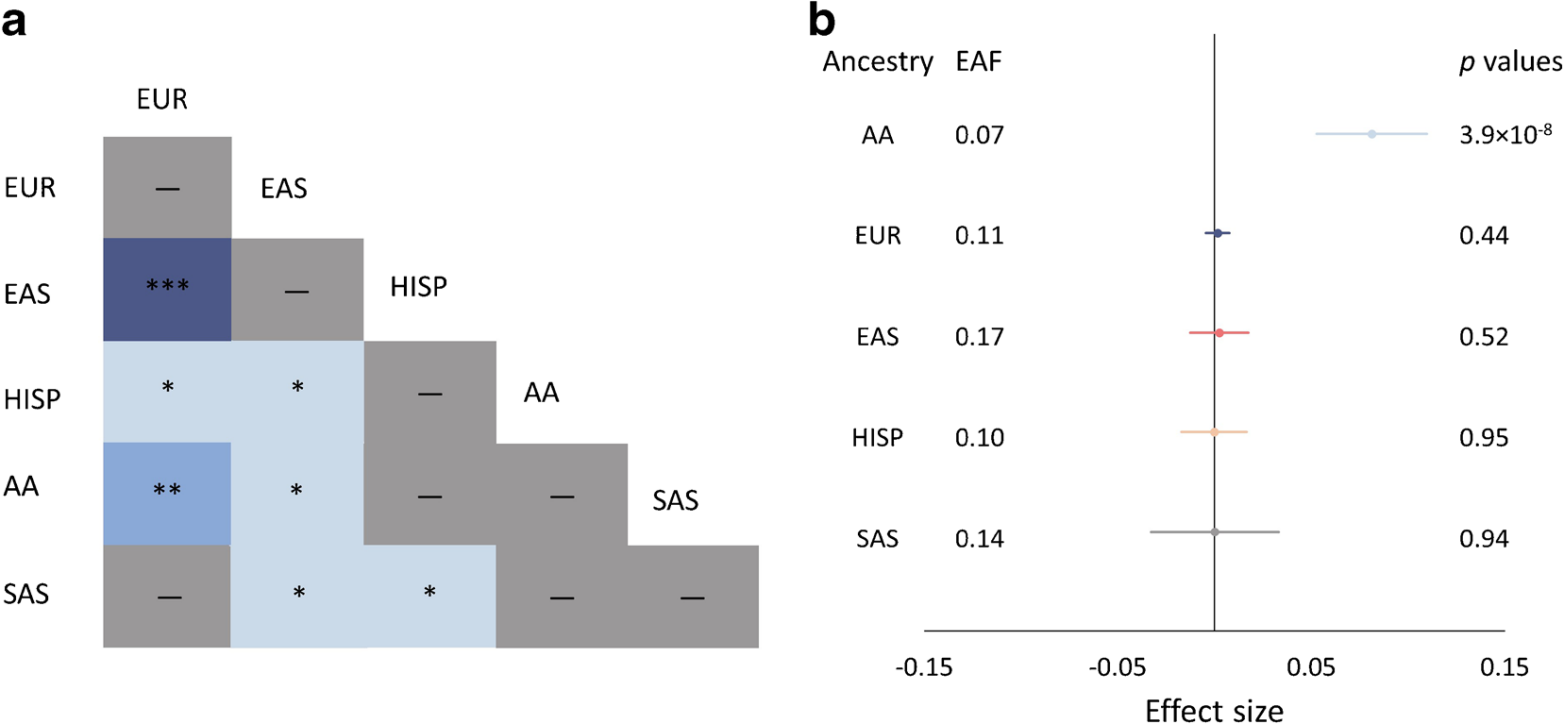
Fasting glucose lead variants with evidence of effect allele heterogeneity across populations of different ancestry. (**a**) Fasting glucose-associated lead variants were tested for evidence of effect allele heterogeneity between populations. The findings from the test of effect allele heterogeneity are shown; a one-side heterogeneity test without multiple testing corrections was conducted and different shades of blue represent different *p* value thresholds (the darker the shade of blue, the more significant the *p* value). *p<1×10^**−**4^ to ≤0.05; **p<1 ×10^**−**6^ to ≤1×10^**−**4^; ***p≤1×10^**−6**^ (dash [–] represents *p*>0.05). (**b**) Forest plot showing the effect allele frequency, effect size, 95% CIs and *p* value for rs61909476, the lead variant associated with fasting glucose in participants of African American ancestry. The same variant shows no evidence of association with fasting glucose in the other ancestries included in the analyses. AA, African American ancestry; EAF, effect allele frequency; EAS, East Asian ancestry; EUR, European ancestry; HISP, Hispanic ancestry; SAS, South Asian ancestry. Adapted from [6]. This figure is available as part of a downloadable slideset

Evidence to date suggests that most common variants associated with type 2 diabetes or continuous glycaemic traits are shared and have broadly equivalent effects across ancestries [6, 33]. However, the Population Architecture using Genomics and Epidemiology (PAGE) Consortium showed significant effect size attenuation at established loci in non-Europeans. As effect sizes were differentially attenuated between ancestries (by ~56% in African Americans and ~24% in Hispanics/Latinos), this suggested the attenuation was not just due to ‘winner’s curse’ [34]. Recently, in a large multi-ancestry meta-analysis, we also found evidence of effect size heterogeneity between populations, in approximately 20% of loci associated with glycaemic traits [6]. For example, we detected significant evidence of effect allele heterogeneity at fasting glucose lead variants between European and East Asian ancestry participants (Fig. 1a). In addition, we found novel loci that had broadly similar allele frequency but with significant effect size differences across ancestries and evidence of association at single ancestries. The variant rs61909476, near *ETS1*, is associated with fasting glucose in African American individuals but not in those from any of the other ancestries, despite broadly similar allele frequency across ancestries (Fig. 1b) [6]. Effect size differences between ancestries can occur because the variant is tagging a causal variant more strongly in one ancestry or because there are population-specific genetic epistatic effects (i.e. genotype-by-genotype interactions) or genotype-by-environment interactions.

### Benefits and challenges of multi-ancestry studies

**Fig. 2:**
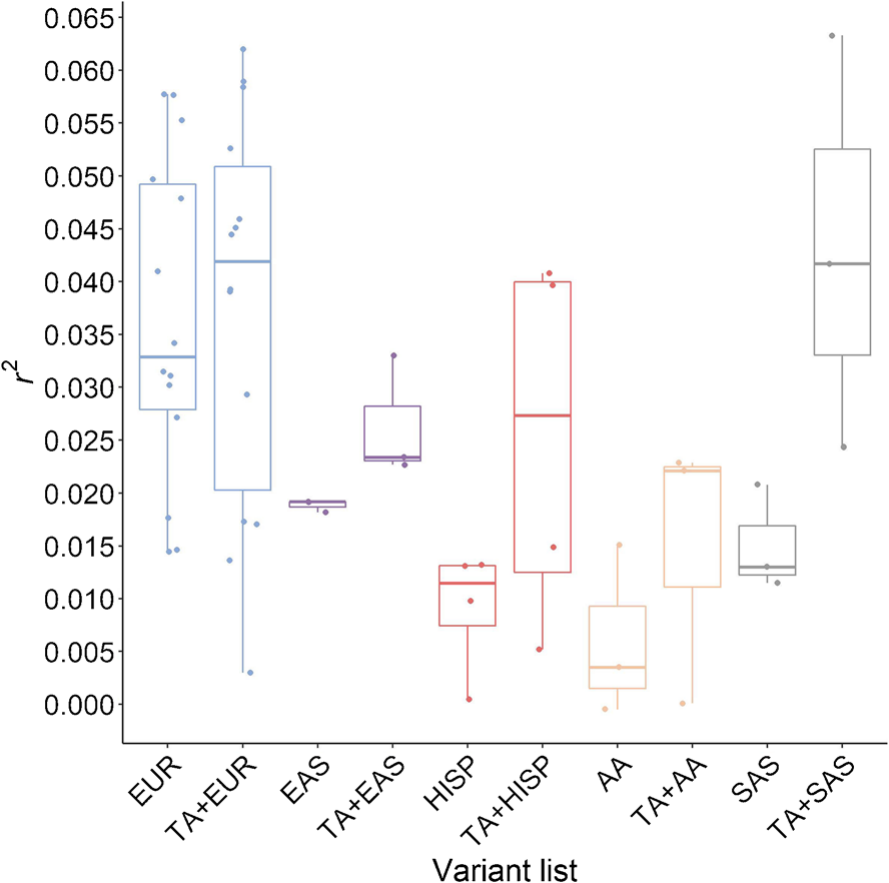
Variance in fasting glucose explained by associated fasting glucose loci. The box and whisker plot shows the trait variance (*r*^2^) explained when using a genetic score with variants that are associated with fasting glucose at genome-wide significant thresholds in each individual ancestry (European [EUR;], East Asian [EAS;], Hispanic [HISP;], African American [AA;] or Southeast Asian [SAS;]), or when using a combination of lead variants identified by meta-analysis across all participating ancestries (trans-ancestry [TA]) and individual ancestry genome-wide associated variants (TA+EUR, TA+EAS, TA+HISP, TA+AA and TA+SAS). Variance in fasting glucose explained by each of the variant lists in each individual ancestry is shown in blue (EUR), purple (EAS), red (HISP), orange (AA) and grey (SAS). The line within each box represents the median, and the top and bottom of the box represent the first and third quartile, respectively. The whiskers indicate the maximum and minimum values. Data points represent the variance explained in individual cohorts used in the analysis. Figure from [6]. This figure is available as part of a downloadable slideset

Multi-ancestry approaches have improved global representation, vastly increased the total sample size of type 2 diabetes and related quantitative trait genetic studies, and have yielded additional associated loci that have effects across populations from multiple ancestries. Often the variant identified from combined multi-ancestry analysis does not meet stringent genome-wide significance thresholds in individual contributing ancestries but there is still evidence that it captures a proportion of the heritability of that trait in that ancestry (Fig. 2) [2, 4–6]. Specifically, a recent study by the Meta-Analysis of Glucose and Insulin-related traits Consortium (MAGIC), which included 30% non-European ancestry participants, showed that including lead variants identified from the meta-analysis across ancestries in a genetic score captured more of the trait variance than the more limited set of variants that met stringent genome-wide significant thresholds in that population (Fig. 2) [6].

Challenges of genetic meta-analyses across ancestries relate to differences in linkage disequilibrium (LD) between populations of diverse ancestry. In this setting, clumping of variants into loci cannot be done by LD but rather by grouping together variants based on a predefined distance to the lead variant. One of the challenges of combining diverse population data is that the lead variant can vary between ancestries. Interpreting this can be difficult as it could result from random fluctuation (e.g. slightly different samples with good quality genotype data at each variant) or different tagging of the underlying causal variant, or it could reflect allelic heterogeneity.

### Fine-mapping

The high degree of LD in European populations is both an advantage and disadvantage when conducting GWAS. High LD between variants is beneficial when conducting locus discovery as many correlated variants can point to a strong association signal. However, a consequence of this is that many variants are indistinguishable from one another in terms of their association with a disease/trait and it can therefore be difficult to establish which is the variant(s) driving the association (causal variant[s]). Fine-mapping is improved through increasing sample size so that LD can be ‘broken’ and smaller sets of variants can be identified. An approach which has gained interest is the use of populations of diverse ancestry to refine association signals [35]. Given that the LD structure differs between populations of different ancestry, this can be leveraged to refine association signals and reduce the number of variants that need to be considered as possibly causal. This has facilitated researchers in resolving association signals to identify a smaller number of likely causal variants that can more reasonably be experimentally tested for functional effects [5, 6, 35–37].

However, fine-mapping across ancestries assumes no allelic heterogeneity at the locus being fine-mapped and assumes the causal variant(s) is shared across all populations used. Consequently, where there is true allelic heterogeneity fine-mapping across ancestries may fail. In addition, there may be technical challenges as many methods relying on summary statistics require that all variants used in the fine-mapping step have data from broadly similar sample sizes, otherwise they may identify false-positive causal variants. Moreover, removal of variants due to quality control issues could inadvertently remove the true causal variant. Nonetheless, fine-mapping methods may still identify a set of variants with high probability of being causal, which may lead researchers to follow an incorrect set of variants in downstream analyses. Comparing results from the fine-mapping to the original meta-analysis within and across ancestries is therefore key to ensure that the lead variant(s), for example, are still within the set of likely causal variants after fine-mapping. Fine-mapping across ancestries can also be challenging, as many methods are not able to account for the heterogeneity in LD across ancestries. An important challenge is that different fine-mapping methods will yield different results so, ultimately, functional validation is required to validate causal variants. Finally, phenotype heterogeneity could also underlie some differences across populations. This is less pertinent to quantitative trait measures that are well standardised but can complicate interpretation in disease studies if cases are ascertained based on very different criteria.

## The importance of conducting studies across multiple ancestries for precision medicine

In contrast to existing approaches to medicine that have been described as ‘one size fits all’, precision medicine proposes to take into account individual differences in genetic makeup, environment and lifestyle when considering disease presentation, diagnoses, treatment and prevention [38].

Historically, there has been limited representation of individuals of diverse ancestry in biobanks, in clinical trials [39] and, as discussed earlier, in genetic studies. Lack of representation in studies means that diagnostic thresholds, treatment regimens and prediction models do not consider genetic differences between ancestries. This means that most of the health and economic benefit from genetics-driven approaches to medicine will inequitably benefit higher income countries (and within those, individuals of European descent), increasing health disparities between diverse populations [40].

### Impact of individual variants on diagnosis, treatment response and adverse drug reactions

In addition to the *TBC1D4* nonsense mutation, discussed above, which may have important implications for diabetes diagnosis in Inuit populations, there are other examples of ancestry-differentiated variants with impact on type 2 diabetes diagnosis and treatment.

The *G6PD* Val98Met (rs1050828) variant causes glucose 6-phosphate dehydrogenase deficiency, a haemolytic anaemia that is often silent in carriers (i.e. they may not know they have the mutation). The same variant reduces HbA_1c_ levels (β = −0.81% [95% CI 0.66, 0.96] per minor allele) independently of glucose levels and potentially leads to under-diagnoses of diabetes in carriers [41, 42]. Other *G6PD* variants that lower HbA_1c_ levels have been identified in Hispanic/Latino [43] and Asian populations [44]. In addition, carriers of the α^−3.7^ thalassemia deletion [25] and asymptomatic individuals with sickle cell trait (rs334 Glu7Val) [43, 45] all have reduced HbA_1c_ levels, independent of glucose levels.

Some of the above variants are common (minor allele frequency >10%) in populations with endemic malaria, as they provide protection against severe malaria [46–48], and all affect the utility of HbA_1c_ as a diagnostic test for diabetes in those populations. Because the prevalence of these variants differs between ancestral groups, ignoring genotype at these variants could exacerbate health disparities. In addition, in carriers being treated for diabetes, physicians may overestimate the degree of glucose control (as carriers will have disproportionately low HbA_1c_ for their blood glucose levels) and therefore undertreat [44].

Beyond effects on diagnosis and treatment targets, the *G6PD* Val98Met variant is associated with significant risk of haemolysis in women treated with the antimalarial agent primaquine [49] and the US Food and Drug Administration has declared the need to consider *G6PD* status for patients prescribed certain sulfonylureas [50], highlighting the importance of knowing genotype at this site before prescribing drugs.

### The promise of genetic risk scores

Variants that associate at genome-wide significant levels with a trait or a disease can be used to construct genetic risk scores (GRSs) that explain or predict a certain proportion of the trait variance in the population [51, 52]. The hope is that these scores may have clinical utility by facilitating identification of individuals at higher risk of disease, aiding in differential diagnoses, better targeting of treatment and therapy dosage to patients, and helping to avoid adverse drug reactions.

Early type 2 diabetes GRSs were built on a limited set of variants, explained a relatively modest fraction of phenotypic variance and were not very useful for disease prediction [53–56]. Additionally, as they were mostly built on results from large meta-analyses of European ancestry GWAS, they missed the effects of other ancestry-specific trait-associated variants, namely variants under different types of selection in populations exposed to different environments. Furthermore, the effect size estimates used were overinflated due to ‘winner’s curse’ in discovery studies [57].

However, as sample sizes increased, more variants have been detected that capture more of trait variance. In addition, when genome-wide associated variants from multi-ancestry studies are used to build GRSs, they capture a larger fraction of phenotypic variance than ancestry-specific GRSs even if the variants are not associated with the disease at genome-wide significance level in all ancestries [5, 6, 58]. This suggests that such multi-ancestry efforts may be required for GRSs to be more globally transferable.

### A type 1 diabetes GRS with clinical utility

Provision of the correct diabetes diagnosis is important, as the optimal treatment is different for type 1 diabetes, type 2 diabetes and other rare monogenic forms of diabetes. Here, a type 1 diabetes GRS (and subsequent successor) has clinical utility, improving newborn screening and supporting classification of adult incident diabetes in individuals of European ancestry [59, 60]. It also helps differentiate between type 1 diabetes and monogenic neonatal diabetes or MODY [61] and monogenic autoimmune diabetes [62]. Despite early concerns regarding the transferability of the GRS to other ancestries [59], the GRS was shown to discriminate between monogenic and type 1 diabetes in Iranian children [63]. It also discriminates between type 1 and type 2 diabetes in India, where misclassification of type 1 diabetes and type 2 diabetes is common in young adults due to the high prevalence of early-onset type 2 diabetes at lower BMI [64].

### Polygenic risk scores

Beyond genome-wide significant variants, models that include additional loci in the genome that have not reached this stringent threshold capture a larger fraction of trait variance [65–67]. These variants have been included in polygenic risk scores (PRSs), which are built on a large number of variants in the genome (in the order of thousands to several million), to improve disease prediction [68–70] (see Text box: Concerns regarding transferability of PRSs). 
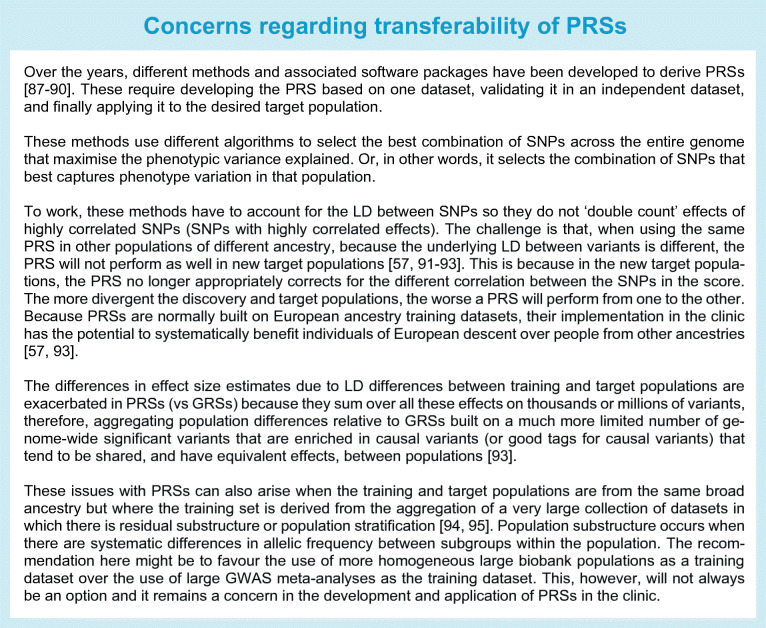


A proposed potential benefit of PRSs is their ability to identify high-risk individuals from birth before classical clinical and biomarker risk factors can be detected, thus enabling identification of a subset of the population who would most benefit from careful screening and monitoring, and from being placed on available preventative strategies or therapies [56]. They may also identify individuals at much larger risk of disease who might not display classical clinical risk factors and would, hence, be missed by current approaches [56], though this has been questioned [71].

Individuals at the top end of the distribution for type 2 diabetes PRSs have a disease risk similar to that of individuals harbouring some monogenic mutations [8, 56]. Nevertheless, concerns regarding portability of these scores across populations (see Text box: Concerns regarding transferability of PRSs) raise doubt over their current clinical utility, and provide a compelling argument for developing scores based on discovery data from diverse populations, as these more readily transfer from discovery to different target populations [57, 58]. Given these concerns, at least for type 1 and type 2 diabetes, it has been suggested by some that GRSs (especially those arising from multi-ancestry analyses) may be currently preferable, as the additional variants in the PRS do not significantly improve the performance of these scores for clinical use [71, 72].

Beyond the cross-population transferability issues that may or may not be addressed by further methodological development, questions remain regarding how PRSs predict disease risk across the lifespan [73, 74], how risk is understood and communicated by practicing clinicians to their patients and, more generally, how to incorporate their use into routine clinical practice [75]. Indeed, the debate rages on as to whether these scores will provide broad clinical utility beyond a few examples [68, 71, 76, 77].

### Partitioned genetic scores

In addition to the use of GRSs and PRSs for disease classification and prediction, the development of partitioned genetic scores corresponding to variants predicted to affect disease through different physiological pathways has gained interest as a means to acquire insight into disease heterogeneity [78, 79]. These partitioned scores may be able to identify subsets of individuals with type 2 diabetes having different risks of complications [79]. Possible clinical utility could additionally result from patient stratification for correct treatment and therapy dosage according to the major pathway predicted to be affected in the subset of patients, and for the identification of participants for clinical trials [56]. However, whether patient stratification for treatment will follow the success seen in monogenic diabetes remains a big question in the field [72].

## Conclusions

**Fig. 3:**
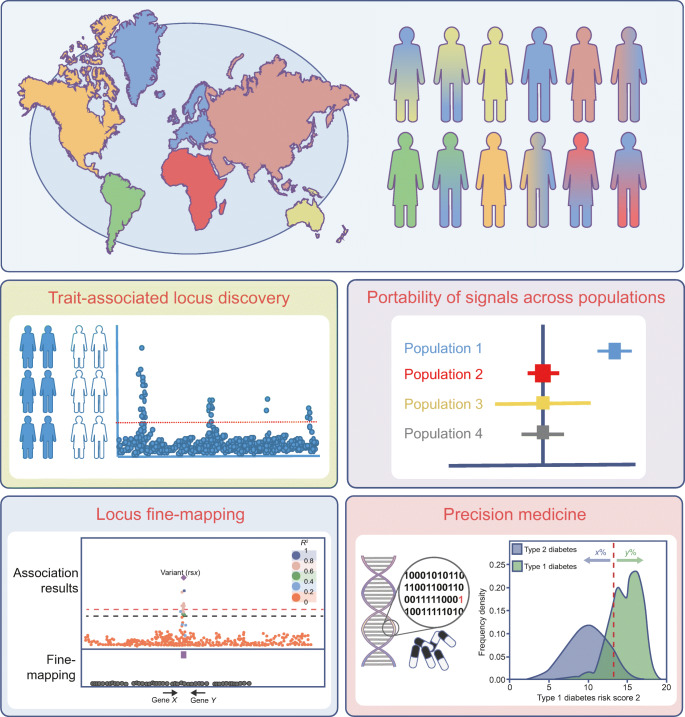
Population diversity in genetic studies of type 2 diabetes and related glycaemic traits. The diagram shows a pictorial representation of the world, with its populations and their admixture represented by the shaded people; the different colours represent differences in ancestral admixture in different individuals. The main areas that benefit from increasing population diversity in genetic studies are shown; these include: trait-associated locus discovery; portability of signals across populations; improving fine-mapping resolution; and development of a more equitable precision medicine approach (e.g. through development of GRSs or PRSs based on multi-ancestral population data). This figure is available as part of a downloadable slideset

Over the last few years, diabetes and glycaemic trait GWAS have included data with broader genetic diversity. This has led to novel locus discovery, improved understanding of the genetic architecture of diabetes and related glycaemic traits across ancestries, improved fine-mapping resolution and resulted in the development of GRSs that better capture disease risk across populations (Fig. 3). Nevertheless, efforts to increase global representation in genetic studies need to be intensified to fully capture the aetiology of type 2 diabetes and associated traits across the world, specifically in under-represented populations, wherein the rise in diabetes prevalence is predicted to be especially notable in the forthcoming years. There is a need to increase representation of different ancestries in regulatory annotation efforts (e.g. generation of expression quantitative trait [eQTL] data), to enable ancestry-specific effects to be interpreted within local context. These annotations have been instrumental in pinpointing causal genes at GWAS loci [8] and are key in the journey from genetic association to improved mechanistic insight.

The opportunities afforded by increasing diversity in genetic studies of type 2 diabetes and related glycaemic traits are undisputed. However, it is important to stress that the human population is a continuum with no discrete boundaries between groups, whether these are defined on the basis of self-reported ethnicity or on the basis of genetically defined ancestry. It is critical therefore, that we move away from describing ancestry based on large continental labels and acknowledge the finer-grained population-level genetic diversity that reflects population history, migration and admixture. Though there are practical reasons for grouping individuals into clusters, in the end we are all admixed with different degrees of contribution from various ancestral groups.

Though significant progress has been made, there remain methodological challenges relating to allelic, phenotypic and environmental heterogeneity. Most importantly, there are significant ethical, societal and cultural challenges still to overcome. Given historical malpractices [80, 81], some communities have naturally become disengaged and suspicious of genetic and genomic efforts. Going forward, engaging with global and indigenous populations needs to be done sensitively and be respectful of local cultures. Ownership of the research agenda and leadership has to be held by those within those communities [82]. An example is the H3A initiative, which set out to empower African researchers to lead and take centre stage in genomic research [83–85]. A considered balance needs to be achieved between the desire to rapidly, publicly, share data globally for the advancement of science and the need to consider critical aspects of indigenous governance policies for self-determination with respect to genomics issues [81]. In addition, equity of access, and ability to use the samples collected and analyse the data generated are important to help level out the playing field [86].

It must also be recognised that for global collaborations between high-income and low- and middle-income countries to be effective, one must take the view of the importance of long-term deliverables rather than focus exclusively on short-term gains. Investment must be made in infrastructure, in building local research capacity and leadership, and in creating opportunities for ‘brain gain’. New initiatives that perhaps focus on bringing experts in from outside for periods of time to conduct research locally, collaborate, train and build local capacity instead of taking local researchers or samples out may avoid so-called ‘helicopter’ or ‘parachute’ science [80, 81]. However, it must be recognised that progress will take time and will need to leverage outside funding to generate investment from local governments. In sum, the road ahead may be long and arduous but it will surely lead us to a better world.

## Supplementary information

Slideset of figures (PPTX 566 kb)

## Abbreviations

GRS: Genetic risk score
GWAS: Genome-wide association studies
LD: Linkage disequilibrium
PRS: Polygenic risk score
