# Supplementary material for: The importance of increasing population diversity in genetic studies of type 2 diabetes and related glycaemic traits
Source: Diabetologia. 2021 Sep 30;64(12):2653–64. doi: 10.1007/s00125-021-05575-4 (PMC8563561; doi:10.1007/s00125-021-05575-4)
Supplement: Supplementary file 1 — (PPTX 566 kb) [file 125_2021_5575_MOESM1_ESM.pptx]

## Slide 1
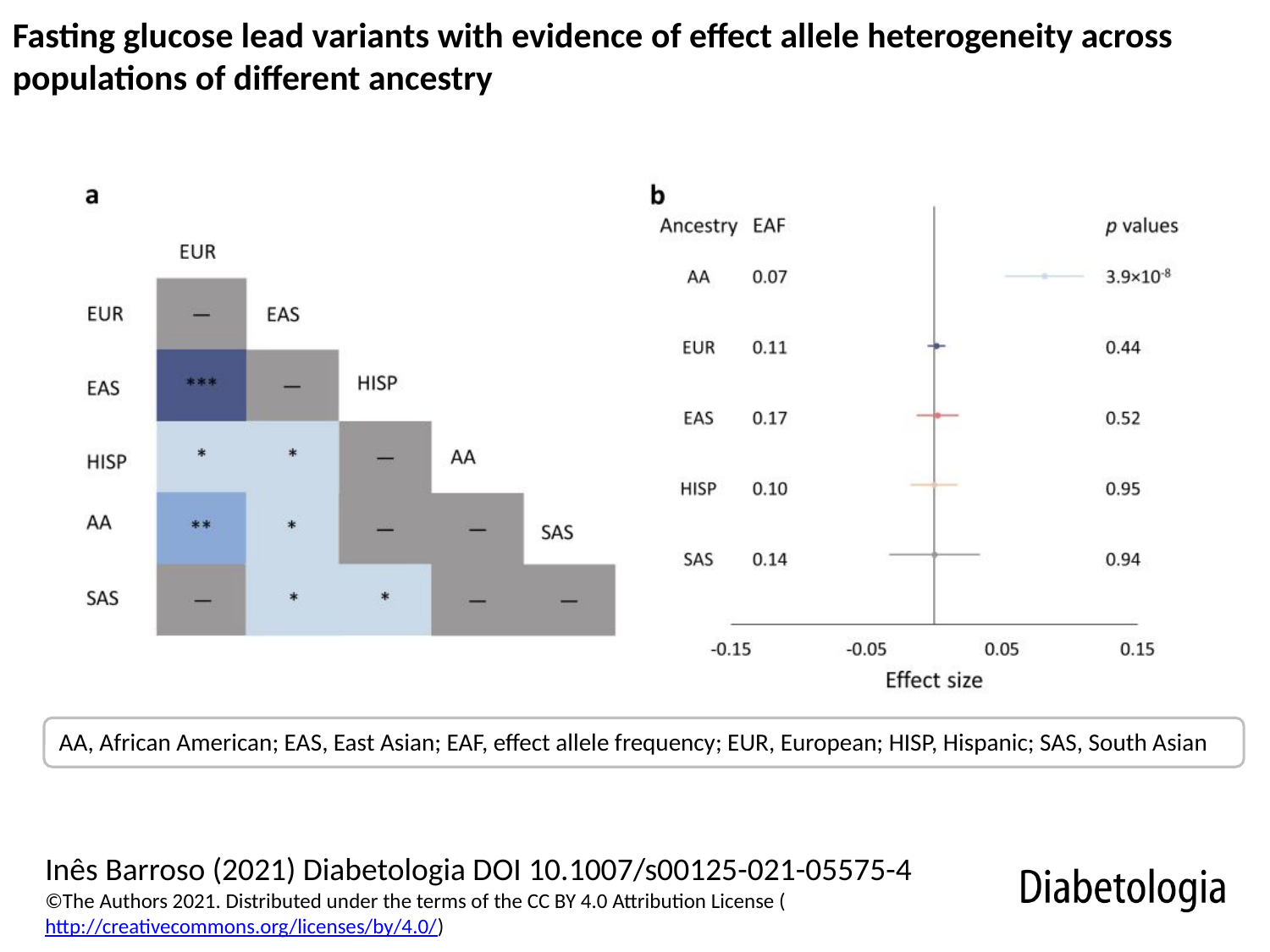

Fasting glucose lead variants with evidence of effect allele heterogeneity across populations of different ancestry
AA, African American; EAS, East Asian; EAF, effect allele frequency; EUR, European; HISP, Hispanic; SAS, South Asian
Inês Barroso (2021) Diabetologia DOI 10.1007/s00125-021-05575-4
©The Authors 2021. Distributed under the terms of the CC BY 4.0 Attribution License (http://creativecommons.org/licenses/by/4.0/)

## Slide 2
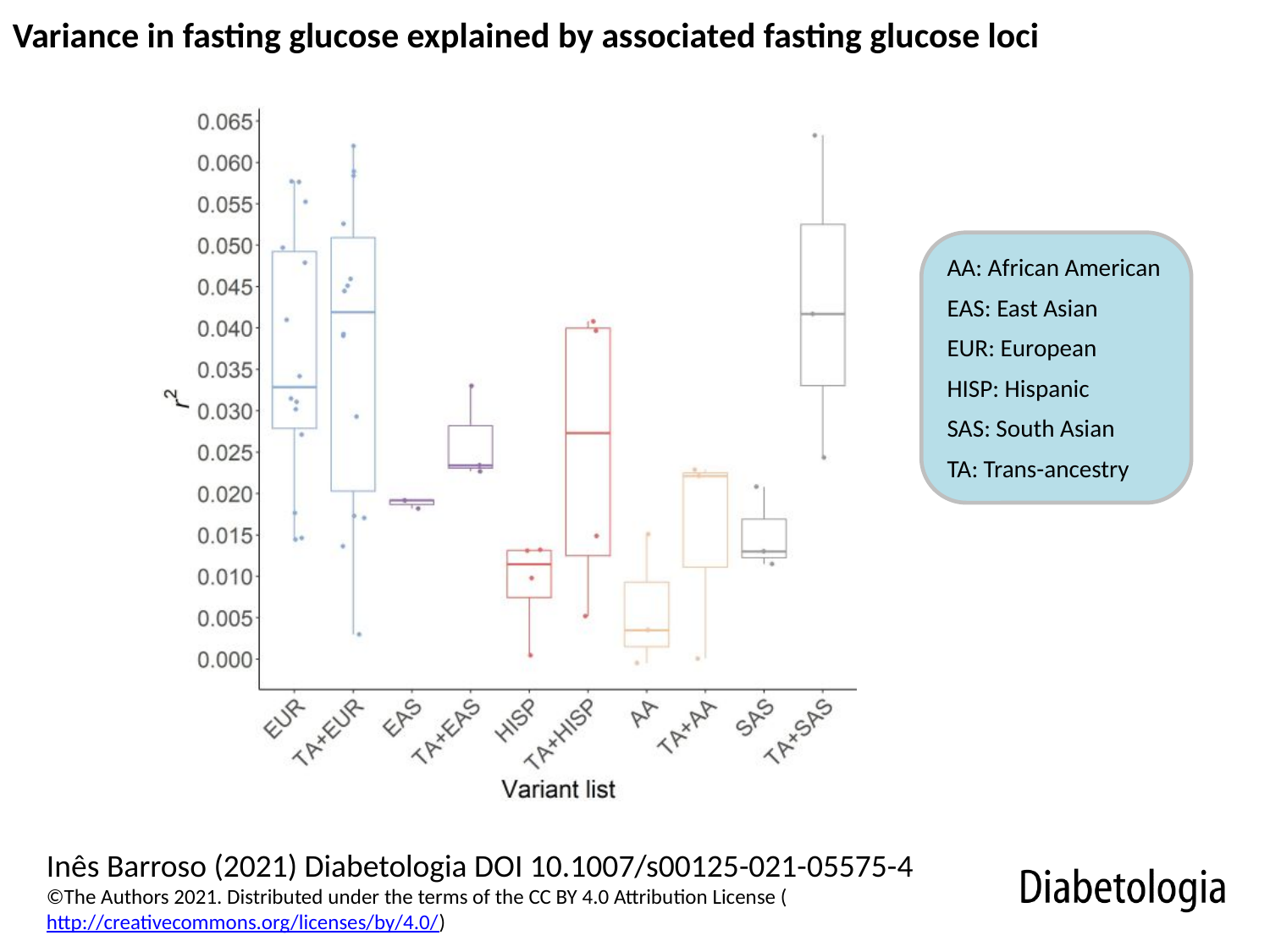

Variance in fasting glucose explained by associated fasting glucose loci
AA: African American
EAS: East Asian
EUR: European
HISP: Hispanic
SAS: South Asian
TA: Trans-ancestry
Inês Barroso (2021) Diabetologia DOI 10.1007/s00125-021-05575-4
©The Authors 2021. Distributed under the terms of the CC BY 4.0 Attribution License (http://creativecommons.org/licenses/by/4.0/)

## Slide 3
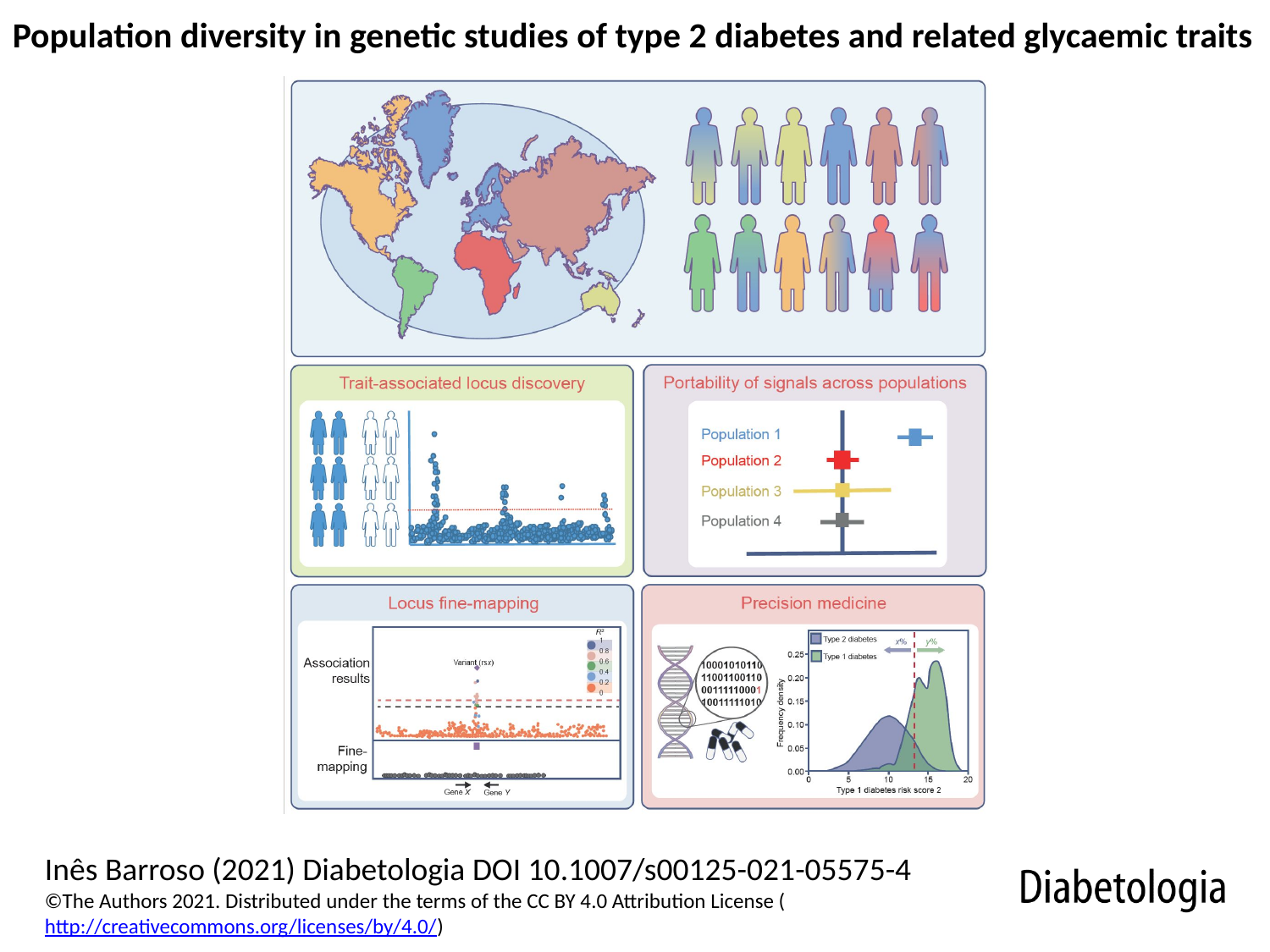

Population diversity in genetic studies of type 2 diabetes and related glycaemic traits
Inês Barroso (2021) Diabetologia DOI 10.1007/s00125-021-05575-4
©The Authors 2021. Distributed under the terms of the CC BY 4.0 Attribution License (http://creativecommons.org/licenses/by/4.0/)
